# A Zn^2+^-triggered two-step mechanism of CLIC1 membrane insertion and activation into chloride channels

**DOI:** 10.1242/jcs.259704

**Published:** 2022-08-03

**Authors:** Lorena Varela, Alex C. Hendry, Joseph Cassar, Ruben Martin-Escolano, Diego Cantoni, Felipe Ossa, John C. Edwards, Vahitha Abdul-Salam, Jose L. Ortega-Roldan

**Affiliations:** 1 School of Biosciences, University of Kent, Canterbury CT2 7NJ, UK; 2 Medway School of Pharmacy, The Universities of Kent and Greenwich at Medway, Chatham ME7 4TB, UK; 3 Centre for Cardiovascular Medicine and Device Innovation, William Harvey Research Institute (WHRI), Faculty of Medicine and Dentistry, Queen Mary University of London, Room 213, John Vane Science Centre, Charterhouse Square, London EC1M 6BQ, UK; 4 Department of Internal Medicine, St Louis University, 3635 Vista Ave., St Louis, MO 63110, USA

**Keywords:** Membrane protein, Chloride channel, Channel activation, Lipid–protein interaction, Biophysics

## Abstract

The chloride intracellular channel (CLIC) protein family displays the unique feature of altering its structure from a soluble form to a membrane-bound chloride channel. CLIC1, a member of this family, is found in the cytoplasm or in internal and plasma membranes, with membrane relocalisation linked to endothelial disfunction, tumour proliferation and metastasis. The molecular switch promoting CLIC1 activation remains under investigation. Here, cellular Cl^−^ efflux assays and immunofluorescence microscopy studies have identified intracellular Zn^2+^ release as the trigger for CLIC1 activation and membrane insertion. Biophysical assays confirmed specific binding to Zn^2+^, inducing membrane association and enhancing Cl^−^ efflux in a pH-dependent manner. Together, our results identify a two-step mechanism with Zn^2+^ binding as the molecular switch promoting CLIC1 membrane insertion, followed by pH-mediated activation of Cl^−^ efflux.

## Abstract (precis)

**Summary:** Identification of a two-step mechanism of CLIC1 membrane insertion based on Zn^2+^ binding and pH activation of Cl^−^ efflux.

## INTRODUCTION

The chloride intracellular channel (CLIC) family consists of a group of highly homologous human proteins with a striking feature, their ability to change their structure upon activation from a soluble form into a membrane-bound chloride channel, translocating from the cytoplasm to intracellular membranes (Tulk et al., 2000; Valenzuela et al., 1997). CLIC1 is the best characterised of the CLIC protein family. It is expressed intracellularly in a variety of cell types, being especially abundant in heart and skeletal muscle (Valenzuela et al., 1997). The integral membrane form of CLIC1 has been found to be localised mostly in the nuclear membrane, although it is present in the membranes of other organelles and transiently in the plasma membrane (Valenzuela et al., 2000). It has also been shown to function as an active chloride channel in phospholipid vesicles when expressed and purified from bacteria, showing clear single channel properties (Tulk et al., 2000, 2002).

CLIC1 has been implicated in the regulation of cell volume, electrical excitability (Averaimo et al., 2014), differentiation (Wang et al., 2011), cell cycle (Valenzuela et al., 2000) and cell growth and proliferation (Tung and Kitajewski, 2010). High CLIC1 expression has been reported in a range of malignant tumours (Gritti et al., 2014; Setti et al., 2015; Tian et al., 2014; Wang et al., 2011; Zhang et al., 2015; Zhao et al., 2015) and cardiovascular diseases such as pulmonary hypertension (Abdul-Salam et al., 2010) and ischaemic cardiomyopathy (Gronich et al., 2010). One common effect of CLIC1 in these disease mechanisms is its effect on endothelial function (Gurski et al., 2015; Xu et al., 2016). CLIC1 compromises endothelial barrier function and mediates cell growth, angiogenesis and migration (Knowles et al., 2012; Tung and Kitajewski, 2010). These vascular malformations have been linked to electrophysiological alterations in the cells caused, at least in part, by the CLIC1 chloride channel activity (Gurski et al., 2015). The activity and deleterious function of CLIC1 is modulated by its equilibrium between the soluble cytosolic form and its membrane-bound form. Only CLIC1 in its channel form has been shown to have pro-proliferative and angiogenic activity, and specific inhibition of CLIC1 channel halts endothelial injury, tumour angiogenesis and progression and vascular inflammation.

Despite its clinical importance, to date only conflicting information is available on the mechanism of CLIC1 membrane insertion, and a high-resolution structure of the channel form has not been determined. *In vitro* oxidation with hydrogen peroxide causes a conformational change due to the formation of a disulfide bond between Cys24 and the non-conserved Cys59, exposing a hydrophobic patch that promotes the formation of a dimer (Littler et al., 2004) in a process that has been proposed to lead to membrane insertion (Goodchild et al., 2009). However, numerous studies have shown that oxidation does not promote membrane insertion (Singh and Ashley, 2006), with evidence pointing at pH (Fanucchi et al., 2008; Stoychev et al., 2009) or cholesterol (Hossain et al., 2016; 2017) as the likely activation factors. Thus, long standing inconsistencies in the data surrounding the molecular switch that transforms CLIC1 from its soluble form into a membrane-bound channel, and the complex nature of its regulation, have prevented further advances in the understanding of CLIC1 function.

In this study, we have explored the membrane insertion activation mechanism of CLIC1. We have discovered the activation of CLIC1 Cl^−^ efflux by intracellular Zn^2+^ in glioblastoma cells, which also causes CLIC1 relocalisation to the plasma membrane. Finally, *in vitro* studies with purified CLIC1 showed Zn^2+^-driven activation of Cl^−^ efflux, membrane association, as well as CLIC1-specific binding to both Zn^2+^ and Ca^2+^.

## RESULTS

Divalent cation binding, specifically Ca^2+^, is involved in the membrane interactions of other protein families (Dubé et al., 2016; Rosengarth and Luecke, 2003; Sciacca et al., 2013). Interestingly, the CLIC *C. elegans* and *Drosophila* homologs EXC-4 and *Dm*CLIC1 (also known as Clic) both contain a bound Ca^2+^ ion that is trapped during the expression and purification process. We therefore sought to understand if divalent cations may have a role in CLIC1 activation as a chloride channel.

**Fig. 1.**
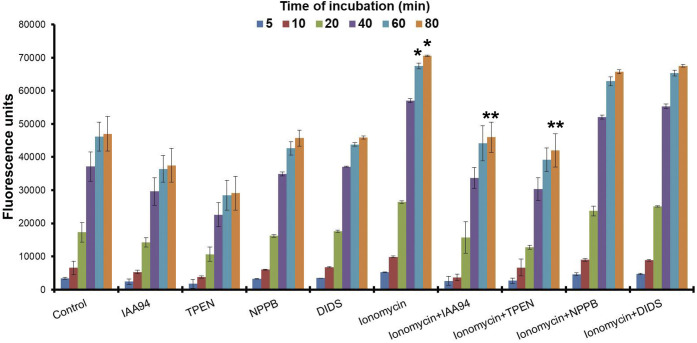
**Effect of different treatments on Cl^−^ efflux.** Results are in fluorescence intensity units for the dye MQAE from MQAE-stained U87G cells exposed to IAA94 (10 µM), TPEN (5 µM), NPPB, DIDS and ionomycin (all 10 µM) for 80 min. Values constitute mean±s.d. of six independent determinations. Ionomycin treatment resulted in statistically significant differences from 60 min compared to the control samples (*P*<0.0123) (*P<0.05) and to all other treatments (**P<0.005) (*n*=6) (two-tailed *t*-test).

The effect of divalent cation-driven membrane localisation on the Cl^−^ efflux activity of CLIC1 was tested in U87G cells using MQAE as a fluorescent reporter of the intracellular concentration of Cl^−^. With no treatment, the Cl^−^ efflux activity is not affected by the chloride channel inhibitors DIDS and NPPB, in line with previous results (Warton et al., 2002), and is only modestly inhibited by the CLIC inhibitor IAA-94 or the Zn^2+^ chelator TPEN, and thus is likely not CLIC mediated. Ionomycin, an ionophore previously shown to increase both Ca^2+^ and Zn^2+^ intracellular concentrations (Ackland and McArdle, 1996), induced a significant increase (*P*=0.0123) of the Cl^−^ efflux, as seen by the increase in MQAE fluorescence, that was reversed upon treatment with the Zn^2+^ chelator TPEN or with the CLIC inhibitor IAA-94 (*P*<0.005) (Xu et al., 2016) (Fig. 1; Fig. S1). Interestingly, TPEN inhibits the Cl^−^ conductance of the control cells to a level similar to that seen upon IAA-94 treatment, suggesting the involvement of Zn^2+^ in the activation of CLIC1 Cl^−^ efflux. Again, DIDS and NPPB showed no effect on CLIC1 efflux after treatment with ionomycin.

**Fig. 2.**
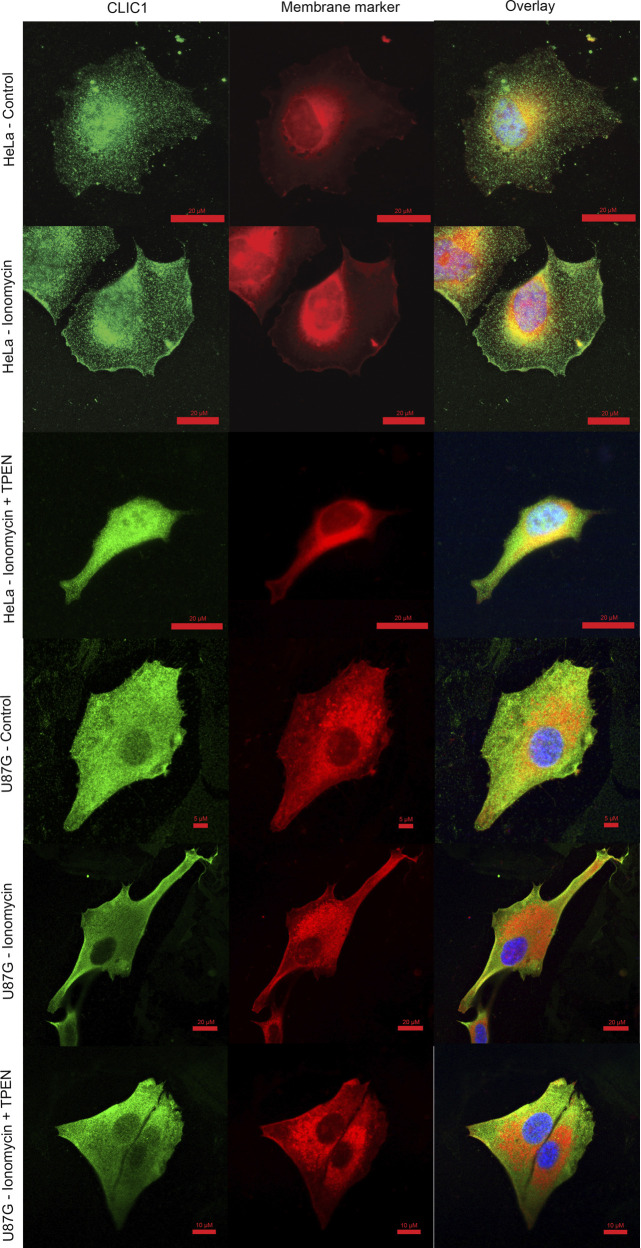
**Divalent cations promote membrane insertion.** CLIC1 localisation in HeLa and U87G cells. HeLa and glioblastoma cells (U87G) were either untreated (1st and 4th row), treated for 3 h with 10 µM ionomycin (2nd and 5th row) or treated with 10 µM ionomycin and the Zn^2+^ chelator TPEN (3rd and 6th row). CLIC1 typically exists in the cytosol and the ionomycin-driven release of Ca^2+^ and Zn^2+^ promoted the presence of CLIC1 at plasma membrane. This effect was reversed upon chelation of intracellular Zn^2+^ by TPEN. Samples were stained with an anti-CLIC1 antibody (green), a CellMASK membrane dye (red) and DAPI (blue). Scale bars indicated in all images correspond to 5–20 µm.

In light of these results, we questioned whether Zn^2+^ could trigger CLIC1 membrane relocalisation in cells. Endogenous CLIC1 localisation was monitored in HeLa and glioblastoma cells (U87G) using immunostaining. CLIC1 typically exists in the cytosol in untreated HeLa cells, and the addition of the ionophore ionomycin promoted the presence of CLIC1 at the plasma membrane (Fig. 2). A similar effect was observed in U87G cells. Addition of ionomycin increased the degree of CLIC1 plasma membrane localisation. For both cell lines, this effect was reversed by the addition of TPEN, confirming that Zn^2+^ triggers the activation and membrane relocalisation of CLIC1.

**Fig. 3.**
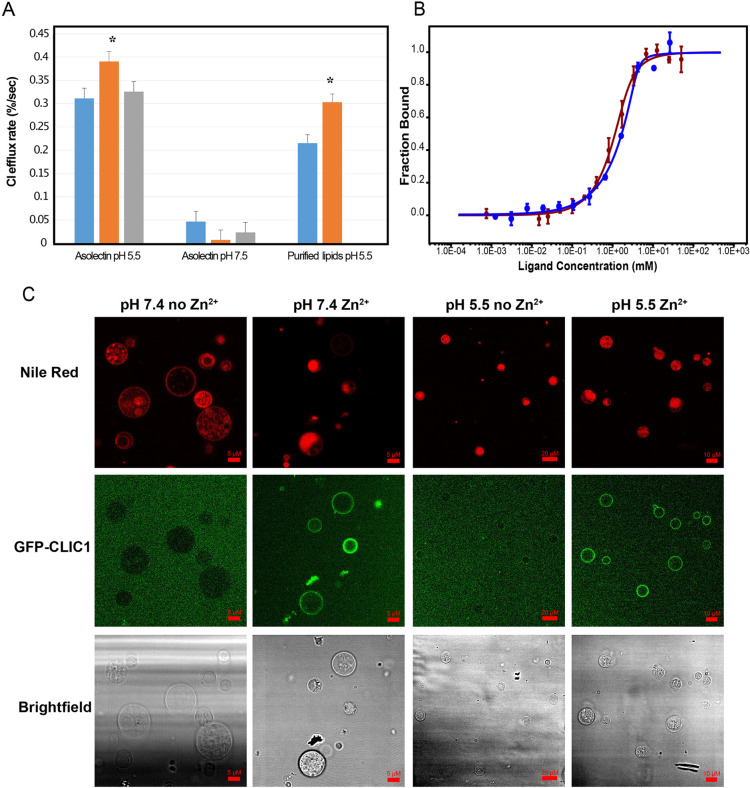
**CLIC1 binds to Zn^2+^, triggering membrane insertion and pH-dependent Cl^−^ conductance.** (A) Zn^2+^ dependence of the CLIC1 Cl^−^ efflux rate. Cl^−^ efflux rates of CLIC1 in the absence of divalent cations (blue) or in the presence of stochiometric amounts of Zn^2+^ (orange) or Ca^2+^ (grey). Results are mean±s.d. (*n*=4). Zn^2+^ treatment resulted in statistically significant differences (*P<0.05, two-tailed *t*-test) compared to the control samples in asolectin vesicles and using purified lipids at pH 5.5. (B) MST binding curves for CLIC1 titrations with Ca^2+^ (red) or Zn^2+^ (blue), displaying a *K*_d_ of 0.3±0.1 mM for Ca^2+^ and 0.7±0.2 mM for Zn^2+^. Results are mean±s.d. (*n*=3). (C) Fluorescence microscopy images of asolectin. Asolectin GUVs labelled with Nile Red dye incubated with GFP-labelled CLIC1 at pH 7.4 (columns 1 and 2) and pH 5.5 (columns 3 and 4) in the absence (columns 1 and 3) and presence (columns 2 and 4) of Zn^2+^. Row 1 displays Nile Red fluorescence, row 2 CLIC1–GFP fluorescence and row 3 shows the brightfield images of the GUVs. Images representative of two experiments. Scale bars indicated in all images correspond to 5–20 μm.

Previous studies have indicated the importance of low pH for CLIC1 chloride channel activity. We used the previously reported vesicle-based Cl^−^ efflux assay to assess effect of either Ca^2+^ or Zn^2+^ ions on CLIC1 chloride channel activity *in vitro* (Tulk et al., 2002). The CLIC1 chloride channel shows strong pH dependence, with little activity above pH 6.5 (Warton et al., 2002). We measured channel activity at pH 5.5 and pH 7.5 in the nominal absence of divalent cations, or in the presence of 1 µM Ca^2+^ or Zn^2+^ (Fig. 3A), using vesicles from the soya bean lipid extract asolectin. This divalent cation concentration was chosen to be equimolar with the protein concentration in the reaction mix. Protein was mixed with cations prior to addition of vesicles. We found virtually no activity at pH 7.5 in the presence or absence of divalent cation. At pH 5.5, we found significant Cl^−^ permeability conferred by CLIC1 that was enhanced ∼25% by the presence of Zn^2+^ (*P*=0.024), but not by the presence of Ca^2+^. We repeated the experiment using purified phospholipid vesicles rather than asolectin because of concern that the activity in the nominal absence of Zn^2+^ might be due to small amounts of Zn^2+^ contaminating the lipids. With purified phospholipids, the activity in the absence of Zn^2+^ was 35% lower than with asolectin vesicles, but the activation with 1 µM Zn^2+^ was greater and amounted to a statistically significant 40% increase (*P*=0.0044).

We subsequently attempted to separate activation of the CLIC1 chloride channel efflux activity from its insertion in the membrane. To this end, we explored the effect of Zn^2+^ and pH on membrane association of recombinantly expressed and purified GFP-tagged CLIC1 by incubating CLIC1–GFP with and without Zn^2+^ at pH 7.4 and 5.5 with giant unilamellar vesicles (GUVs). We used the fluorescence of Red Nile dye, which binds to the GUVs lipids, and GFP, to investigate the degree of colocalisation of CLIC1–GFP with the GUVs. Fluorescence microscopy images collected showed CLIC1 colocalisation in the membrane only in the presence of Zn^2+^ irrespective of the pH value, indicating that Zn^2+^ has a direct effect on CLIC1, triggering its membrane insertion (Fig. 3C). Ca^2+^ on the other hand only induced partial insertion in the membrane at the same protein and cation concentrations, in agreement with its neglectable effect on CLIC1 Cl^−^ efflux (Fig. S2).

Microscale thermophoresis (MST) can be used to identify specific divalent cation binding to CLIC1 and to determine the affinity of these interactions. We used a C-terminal GFP tagged CLIC1 construct at a concentration of 1 µM and at pH 7.4 to compare the binding affinities of Ca^2+^ and Zn^2+^ ions in the presence of asolectin vesicles, as CLIC1 can aggregate at molar ratios of Ca^2+^ and Zn^2+^ higher than 1 in the absence of lipids. CLIC1 showed specific binding to both ions, with Zn^2+^ and Ca^2+^ having a similar apparent affinity at 300–700 µM (Fig. 3B).

## DISCUSSION

### A model for CLIC1 membrane insertion

**Fig. 4.**
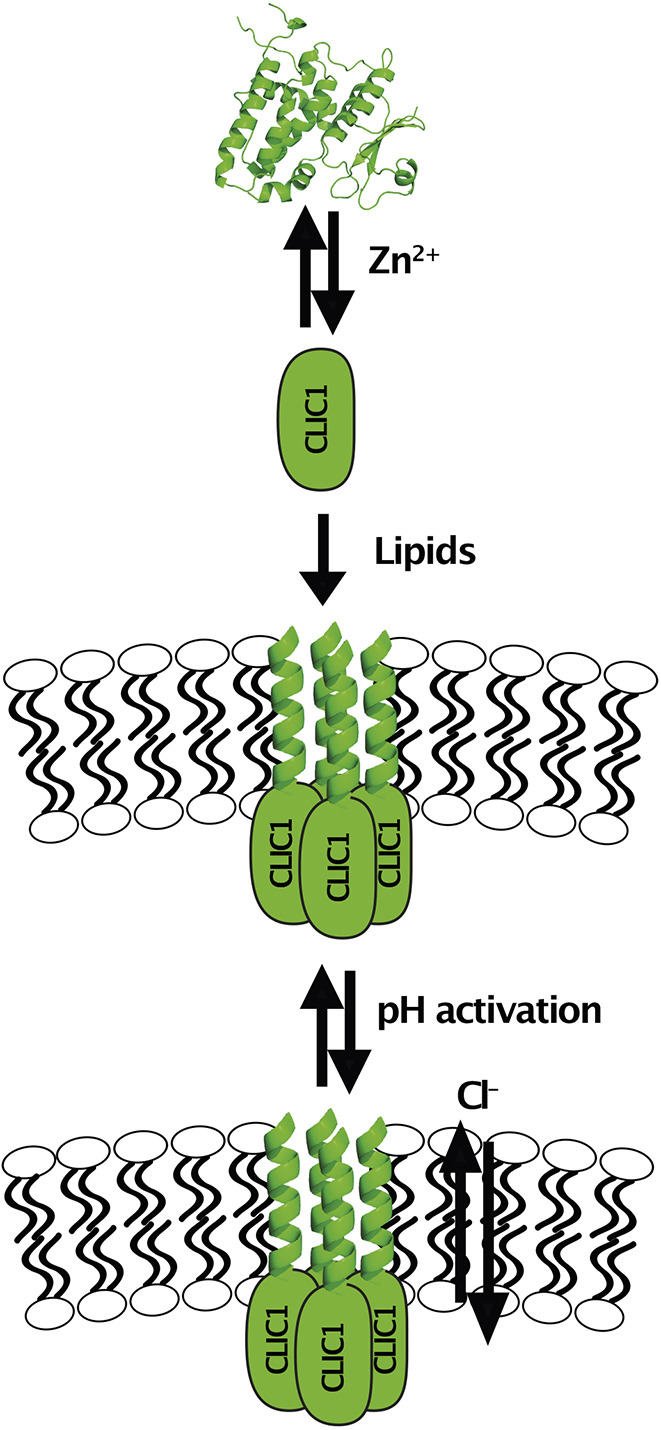
**A two-step model for CLIC1 activation and membrane insertion.** CLIC1 binds to Zn^2+^, inducing a conformational change that triggers membrane insertion. The CLIC1 Cl^−^ efflux activity is then activated at low pH.

In this study, we demonstrate that CLIC1 binds to Zn^2+^, promoting membrane insertion in both model membranes and in a cellular environment. Once inserted, Cl^−^ efflux is only activated at low pH values. Combining our data, we propose a new two-step activation mechanism of CLIC1 whereby CLIC1 exists in a soluble state, and upon intracellular Zn^2+^ release CLIC1 binds to it, alters its structure and inserts into the membrane (Fig. 4). The channel is only activated at low pH, suggesting either a pH gating mechanism or a conformational rearrangement within the membrane at low pH values, since pH had no effect on CLIC1 interaction with the membrane. CLIC1 has been shown to regulate phagosomal acidification (Salao et al., 2016), supporting the role of pH in the activation of the channel.

An increase in the cellular Ca^2+^ and Zn^2+^ concentration has been linked to upstream and downstream changes of the reactive oxygen species (ROS) signalling pathway (Görlach et al., 2015), explaining previous studies on the relationship between CLIC activity and ROS production and oxidative stress. Ionomycin is known to increase both intracellular levels of Zn^2+^ and Ca^2+^ (Ackland and McArdle, 1996). We show that although ionomycin treatment could result in CLIC1 binding to both Zn^2+^ and Ca^2+^ with similar affinity, only Zn^2+^ is able to induce membrane association (Fig. S2) and Cl^−^ efflux (Fig. 3A). This is further supported by treatment with the Zn^2+^ chelator TPEN, which is able to suppress CLIC1 membrane relocalisation and its Cl^−^ conductance. The higher activation potential of Zn^2+^ over Ca^2+^ must therefore be a result of a higher degree of structural changes resulting from the interaction with Zn^2+^. The apparent affinity of CLIC1 towards Zn^2+^ and Ca^2+^ determined by MST is weak, whereas at molar ratios of 1:1 Zn^2+^ significantly increases CLIC1 Cl^−^ conductance. MST relies on the presence of a fluorescent molecule attached to the protein, and in this case, we used a GFP fused to the C-terminus of CLIC1. We hypothesise that the presence of this C-terminal GFP might be interfering with the structural changes that need to occur for CLIC1 to bind to Zn^2+^ and to insert in the membrane, lowering the affinity for this interaction.

Divalent cations have been related to the membrane insertion properties of proteins of the annexin family (Rosengarth and Luecke, 2003), as well as the E1 membrane protein of rubella virus (Dubé et al., 2016) and the amyloidogenic peptide amylin (Sciacca et al., 2013), promoting the interactions of the soluble forms of these proteins with negatively charged lipids. CLIC1 has been shown to have maximal Cl^−^ efflux activity in the extract of soya bean lipid asolectin, which is rich in the lipid phosphatidylethanolamine (PE), phosphatidylcholine (PC) and the negatively charged phosphatidylinositol (PI) classes (Tulk et al., 2002), supporting the role of divalent cations in CLIC1 membrane insertion. Annexin membrane association is expected to occur due to the exposure of an otherwise buried amphipathic segment upon binding to Ca^2+^ ions. CLIC1 contains a region (residues 24–41) with moderate hydrophobicity and a moderate hydrophobic moment that could, in a similar mechanism, detach from the globular structure of the protein upon divalent cation binding, become exposed to the solvent and mediate association with the membranes, likely forming a helix. The hydrophobicity of this segment would also explain the aggregation of the protein at higher concentrations of Ca^2+^ and Zn^2+^ ions in the absence of lipids. Interestingly, the same region in the structurally homologous glutathione S-transferase is not hydrophobic, suggesting that this helix plays a different role in the CLIC family.

Although the structural rearrangements involved in this process are not yet fully understood, the molecular switch between the soluble and membrane-bound forms is now elucidated, complementing the previously known effects of pH and cholesterol. This enables, for the first time, the manipulation of CLIC1 localisation in cellular systems and the recombinant production of CLIC1 samples in the chloride channel form in membrane mimetic systems (Medina-Carmona et al., 2020), and provides a clear mechanism for the channel formation process of this unusual and clinically important protein.

## MATERIALS AND METHODS

### Protein expression and purification

The human *CLIC1* gene (clone HsCD00338210 from the plasmid service at Harvard Medical School) was cloned into a pASG vector (IBA) containing an N-terminal twin strep tag and a TEV cleavage site, and into a pWaldo vector (Drew et al., 2001) containing a C-terminal GFP and a TEV cleavage site. CLIC1 was expressed recombinantly in the C43 *E. coli* strain (Lucigen). The cells were lysed by sonication, and the membrane and soluble fractions were separated by ultracentrifugation using a 70Ti or 45Ti rotor (Beckman) at 117,734 ***g***. Membrane-bound CLIC1 can be extracted using a mixture of 1% DDM (n-dodecyl-β-D-maltopyranoside) (Glycon) and 1% Triton X-100, resulting in solutions with the CLIC1 soluble form. Both fractions were purified separately in the absence of any detergent using affinity chromatography with a Strep-Tactin XT column. The elutions were pooled and cleaved with TEV protease (produced in-house), and subsequently gel filtrated using a Superdex200 Increase column (GE Healthcare) in either 20 mM HEPES buffer with 20 mM NaCl at pH 7.4 or 20 mM potassium phosphate buffer with 20 mM NaCl at pH 7.4.

### Cl^−^ efflux assays

U87G cells (courtesy of Prof. Martin Michaelis, University of Kent, UK) were maintained in Dulbecco's modified Eagle's medium (DMEM) supplemented with 10% fetal bovine serum (FBS) (both Pan-Biotech) and 1% penicillin/streptomycin (Thermo Fisher Scientific) at 37°C, 95% humidity and 5% CO_2_.

For *N*-(Ethoxycarbonylmethyl)-6-Methoxyquinolinium Bromide (MQAE; Thermo Fisher Scientific) assays U87G cells were seeded into dark 96-well microtiter flat-bottom plates at 4×10^4^ cells/well in 100 µl volumes DMEM supplemented with 10% FBS and 1% penicillin/streptomycin and incubated overnight at 37°C, 95% humidity and 5% CO_2_. Cells were then stained with 8 mM MQAE for 2 h (Andersson et al., 2003), and subsequently washed three times with PBS. Non-stained cells were also included. A variety of conditions were evaluated after MQAE loading. In conditions where inhibitors were used, these – 5 µM TPEN or 10 µM IAA-94, DIDS or NPPB (Abcam) – were added and incubated for 10 min. Finally, 10 µM ionomycin was added and repetitive fluorescence measurements (every 1 min for 80 min) were initiated immediately using an Omega fluorescence plate reader (excitation, 355 nm; emission, 460 nm). Six separate wells were used for each biological repeat. The mean±s.d. was calculated for each condition and time.

### Cl^−^ efflux assays with purified CLIC1

CLIC1 chloride channel activity was assessed using the Cl^−^-selective electrode assay described previously (Tulk et al., 2002). Recombinant CLIC1 was purified as previously described (Tulk et al., 2002) except EDTA was included at 1 mM in all steps following the thrombin digestion until the final dialysis change. Unilamellar asolectin or purified lipid (consisting of 80% palmitolyloleolyl phophotidylcholine, 10% palmitoyloleoyl phosphotidylserine and 10% cholesterol) vesicles (Tulk et al., 2002) were prepared at 20 mg/ml in 200 mM KCl, 5 mM HEPES (pH 7.4). CLIC1 protein at 1 µM final concentration was incubated in the presence or absence of divalent cation as indicated in 380 μl volume of 200 mM KCl, and 10 mM MES pH 5.5 or HEPES pH 7.5. 20 μl of lipid suspension were added to the protein mixture with vortexing and incubated for 5 min at room temperature. The lipid mixture was then applied to a 3.5 ml Biogel-P6DG spin column previously equilibrated in 330 mM sucrose. The eluate from the spin column was added to a cup containing 330 mM sucrose, 10 μM KCl, 10 mM MES pH 5.5 or HEPES pH 7.5 as appropriate, with continuous monitoring of extravesicular Cl^−^ concentration with an ion-selective electrode. Valinomycin (Sigma-Aldrich) was added to 1 μM to initiate voltage driven Cl^−^ efflux, followed by Triton X-100 to 1% to release remaining intravesicular Cl^−^. The initial rate of fractional Cl^−^ efflux after addition of valinomycin is taken as the Cl^−^ permeability.

### Fluorescence microscopy

GUV formation was carried out using a protocol adapted from Manley and Gordon (2008) and Veatch (2007). An asolectin lipid stock was prepared in 50 mM HEPES, 50 mM NaCl pH 7.4 buffer. 2 µl/cm^2^ of 1 mg/ml lipid mixed with 1 mM Nile Red lipophilic stain (ACROS Organic) was applied to two ITO slides and dried under vacuum for 2 h. 100 mM sucrose, 1 mM HEPES pH 7.4 buffer was used to rehydrate the lipids in the described chamber. 10 Hz frequency sine waves at 1.5 V were applied to the chamber for 2 h. Liposomes were recovered and diluted into 100 mM glucose, 1 mM HEPES, pH 7.2 buffer. For all four assays, 90 nM CLIC1–GFP was incubated with the GUVs with 0.5 mM ZnCl_2_, 0.5 mM CaCl_2_, or without ions, at room temperature for 10 min. Microscopy for each assay was performed in an 8-well Lab-Tek Borosilicate Coverglass system (Nun) with a Zeiss LSM-880 confocal microscope using 488 nm and 594 nm lasers. All images were processed with Zen Black software.

For immunofluorescence assays, cells were seeded into 24-well plates onto sterile microscopy slides for next day treatment. At 24 h post seeding cells were washed with Tris-buffered saline (TBS), cells were transferred to phosphate-free medium and left untreated or treated with 10 µM ionomycin or 10 µM *N*,*N*,*N*′,*N*′-Tetrakis(2-pyridylmethyl)ethylenediamine (TPEN) as required. Fixation with 4% formaldehyde for 15 min was carried out at 2 h post treatment. The cells were then washed and permeabilised for 10 min with 0.1% Triton X-100 in TBS and washed twice with TBS to remove any detergent. Cells were incubated with CellMASK Deep Red plasma membrane stain (Invitrogen) for 15 min. Following a TBS wash step, the cells were blocked at room temperature with 2% BSA for 1 h. Primary antibody GFP incubation was carried out overnight at 4°C with a 1:50 dilution of monoclonal mouse anti-CLIC1 antibody (Santa Cruz Biotechnology, clone 356.1). After primary incubation, three wash steps were carried out, prior to a 1 h incubation with secondary antibody at a 1:1000 dilution (Alexa Fluor 488-conjugated donkey anti-mouse-IgG, Life Technologies). A further three washes followed, then nucleus staining with NucBlu Live Cell Stain (Invitrogen) for 20 min. The slides were washed a final time and mounted with ProLong Gold Antifade (Invitrogen). All microscopy slides were viewed with a Zeiss LSM-880 confocal microscope using 405 nm, 488 nm and 633 nm lasers. All images were processed with Zen Black and Zen Blue software.

### Microscale thermophoresis

A recombinantly expressed C-terminal GFP tag construct of CLIC1 was used. Titrations were performed at a constant protein concentration of 0.8 µM in HEPES buffer (20 mM HEPES, 20 mM NaCl, pH 7.4) in the presence of 80 µM asolectin vesicles, with increasing concentration of a solution of Zn_2_SO_4_ in the HEPES buffer and a final volume of 20 µl. Prepared samples were filled into Monolith NT.115 capillaries (NanoTemper). Measurements were recorded on a Monolith NT.115 at 30°C, excited under blue light, medium MST power and 15% excitation power. The data were analysed using MO Affinity Analysis software (NanoTemper) and fitted using the *K*_d_ model.

## Supplementary Material

Supplementary information

Reviewer comments
