# Supplementary material for: A Zn2+-triggered two-step mechanism of CLIC1 membrane insertion and activation into chloride channels
Source: J Cell Sci. 2022 Aug 3;135(15):jcs259704. doi: 10.1242/jcs.259704 (PMC9511705; doi:10.1242/jcs.259704)
Supplement: Supplementary information [file joces-135-259704-s1.pdf]

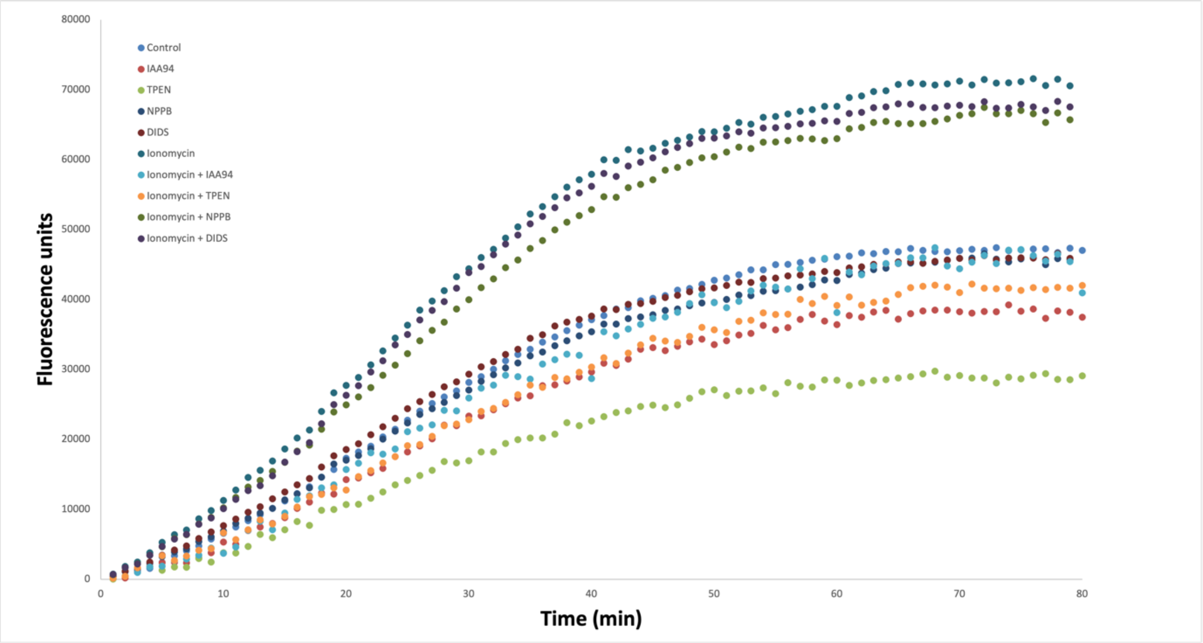

Fig. S1. Effect over time of different treatments on chloride efflux (measured as fluorescence intensity units of the dye MQAE) in MQAE-stained U87 cells exposed to IAA94 (10 µM), TPEN (5 µM), NPPB, DIDS and/Ionomycin (10 µM) for 80 min. Values constitute means of six independent determinations

Journal of Cell Science • Supplementary information

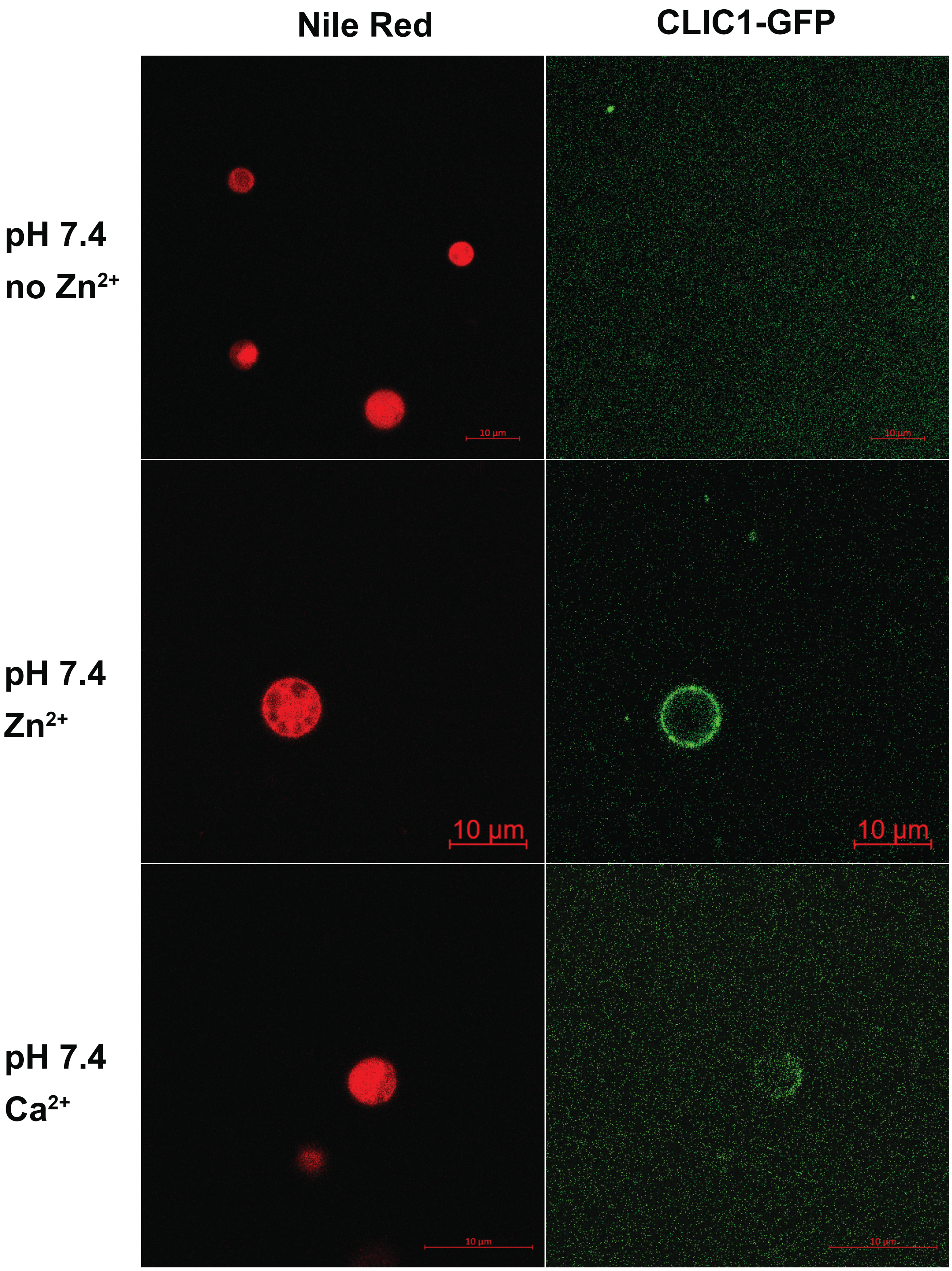

Fig. S2. Fluorescent microscopy images of Asolectin GUVs labelled with Nile red dye incubated with GFP-labelled CLIC1 in the absence of divalent cations and in the presence of 500 µM of Zn^{2+} or Ca^{2+}.

Journal of Cell Science • Supplementary information
