# Supplementary material for: A Zn2+-triggered two-step mechanism of CLIC1 membrane insertion and activation into chloride channels
Source: J Cell Sci. 2022 Aug 3;135(15):jcs259704. doi: 10.1242/jcs.259704 (PMC9511705; doi:10.1242/jcs.259704)
Supplement: Reviewer comments [file joces-135-259704_review_history.pdf]

A Zn^{2+}-triggered two-step mechanism of CLIC1 membrane insertion and activation into chloride channels

Lorena Varela, Alex C. Hendry, Joseph Cassar, Ruben Martin-Escolano, Diego Cantoni, Felipe Ossa, John C. Edwards, Vahitha Abdul-Salam and Jose L. Ortega-Roldan DOI: 10.1242/jcs.259704

Editor: Giampietro Schiavo

Review timeline
Original submission: 19 January 2022
Editorial decision: 16 February 2022
First revision received: 27 June 2022
Accepted: 4 July 2022

Original submission

First decision letter

MS ID#: JOCES/2021/259704

MS TITLE: Zn^{2+} triggered two-step mechanism of CLIC1 membrane insertion and activation into chloride channels

AUTHORS: Lorena Varela, Alex C Hendry, Joseph Cassar, Ruben Martin-Escolano, Diego Cantoni, John C Edwards, Vahitha Abdul-Salam, and Jose Luis Ortega-Roldan
ARTICLE TYPE: Research Article

We have now reached a decision on the above manuscript.

To see the reviewers' reports and a copy of this decision letter, please go to: https://submit-jcs.biologists.org and click on the 'Manuscripts with Decisions' queue in the Author Area. (Corresponding author only has access to reviews.)

As you will see, the reviewers gave favourable reports but raised some critical points that will require amendments to your manuscript. I hope that you will be able to carry these out because I would like to be able to accept your paper, depending on further comments from reviewers.

We are aware that you may be experiencing disruption to the normal running of your lab that makes experimental revisions challenging. If it would be helpful, we encourage you to contact us to discuss your revision in greater detail. Please send us a point-by-point response indicating where you are able to address concerns raised (either experimentally or by changes to the text) and where you will not be able to do so within the normal timeframe of a revision. We will then provide further guidance. Please also note that we are happy to extend revision timeframes as necessary.

Please ensure that you clearly highlight all changes made in the revised manuscript. Please avoid using 'Tracked changes' in Word files as these are lost in PDF conversion.

I should be grateful if you would also provide a point-by-point response detailing how you have dealt with the points raised by the reviewers in the 'Response to Reviewers' box. Please attend to

© 2022. Published by The Company of Biologists under the terms of the Creative Commons Attribution License (https://creativecommons.org/licenses/by/4.0/).

all of the reviewers' comments. If you do not agree with any of their criticisms or suggestions please explain clearly why this is so.

Reviewer 1

Advance summary and potential significance to field

This study adds to the current body of published studies investigating the CLIC family of proteins and their properties as both soluble and membrane proteins.

This manuscript describes interesting findings of a mechanism controlling the insertion of the protein CLIC1 from its soluble form into its integral membrane form. The findings demonstrate the increased recruitment to the membrane of CLIC1 via binding of Zn2+, along with further confirming the pH induced enhancement of its chloride ion transport.

Comments for the author

The manuscript is overall well written and provides sufficient experimental data to support the findings. However, the following points need to be addressed prior to publication.
1. As others in the literature have also reported on alternate mechanisms for the membrane insertion of CLIC1, and there is likely to be more than one mechanism regulating this process, the abstract should be corrected to reflect this. A present the abstract presents these findings as exclusive, which is misleading. Suggested wording shown below:
"Molecular switches promoting CLIC1 activation remain under investigation. Here cellular chloride efflux assays and immunofluorescence microscopy studies have identified Zn2+ intracellular release as a trigger for CLIC1 activation and membrane relocalisation. Biophysical assays confirmed specific binding to Zn2+, inducing membrane association and enhancing chloride efflux in a pH dependent manner. Together, our results identify a two-step mechanism with Zn2+ binding as a molecular switch promoting CLIC1 membrane insertion, followed by pH activation of chloride efflux."

2. The following statement in the introduction should reference (Valenzuela et al., 2000)
"CLIC1's integral membrane form has been found to be localised mostly in the nuclear membrane, although it is present in the membranes of other organelles and transiently in the plasma membrane."
3. Further to point 1, the following statement (and following paragraph) is also not entirely accurate and is simplistic in its argument:
"Despite its clinical importance, to date very little and conflicting information is available for CLIC1 membrane insertion mechanism, and the structure of the channel form is unknown."
Yes the precise structural details of the membrane form of CLIC1 remains unknown however the following references shed light on its membrane insertion and/or membrane structure and there are increasing details regarding various mechanisms regulating its transition from soluble to membrane forms. In particular, the paper by Hossain et al,2017 (Langmuir) included below, provides detailed information regarding the membrane structure determined by X-ray and neutron reflectometry. These more subtle points should be reflected within the manuscript to indicate there is a complex and nuanced regulation processes for CLIC1 membrane insertion.
- Achilonu I, Fanucchi S, Cross M et al., "Role of Individual Histidines in the pH-dependent global stability of Human CLIC1". Biochemistry 2021: 51:995-1004;
- Hossain, Turkewitz, Holt et al., "A conserved GXXXG motif in the transmembrane domain of CLIC proteins is essential for their cholesterol-dependant membrane interaction" BBA - General Subjects 1863 (2019) 1243–125;
- Valenzuela, AlKhamici, Brown et al. "Regulation of the Membrane Insertion and Conductance Activity of the Metamorphic Chloride Intracellular Channel Protein CLIC1 by Cholesterol" PLOS One (Feb 2013) 8:2;
- KR Hossain, SA Holt, AP Le Brun, H Al Khamici, SM Valenzuela "X-ray and neutron reflectivity study shows that CLIC1 undergoes cholesterol-dependent structural reorganization in lipid monolayers" Langmuir 33 (43), 12497-12509.

3. The following final statement is also an overreach with regard to the uniqueness of the findings and should also be modified to take into account the existing literature and the relative place the

© 2022. Published by The Company of Biologists under the terms of the Creative Commons Attribution License (https://creativecommons.org/licenses/by/4.0/).

findings from this study have with respect to the total literature and information currently available regarding CLIC1 and its membrane interactions.
"While the structural rearrangements involved in this process are not yet fully understood, the molecular switch between the soluble and membrane bound forms is now elucidated.
This enables, for the first time, the manipulation of CLIC1 localisation in cellular systems and the recombinant production of CLIC1 samples in the chloride channel form in membrane mimetic systems (Medina-Carmona et al., 2020), and provides a clear mechanism for the channel formation process of this unusual and clinically important protein."

Reviewer 2

Advance summary and potential significance to field

Chloride Intracellular Ion Channel proteins are a novel class of ion channels. One of the CLIC proteins, CLIC1 preferentially localizes to intracellular organelles but on overexpression, they translocate to the plasma membrane. It is not clear how CLICs localize to membranes from the cytosol and whether they conduct Cl through membranes. Authors have shown that Zn not Ca is important for translocation of CLIC1 to the membrane and also increases Cl conductance. The manuscript is important for the field and will influence future work on CLICs.

Comments for the author

The manuscript is focused on understanding the mechanism involved in the localisation of CLIC1 to the membrane. The data presented by the authors to support the role of Zn in CLIC1 to the membrane is convincing but the functional data is not promising.
I have the following comments.
1. CLIC1 is known to localise to the nuclear membrane. In fig. 2, the authors should show colocalization with nuclear and plasma membrane markers. Colocalisation should be quantified.
2. In Fig 2. experiments should also be carried out in presence of ionophore as done for other experiments.
3. The major issue with proposing CLICs as functional ion channels is the time taken for Cl efflux. This is a long-standing argument in the field. In figure 1, it will be helpful to show fluorescence vs time. If CLIC1 is an ion channel, one should observe a faster component in the graph. Alternatively, you can perform electrophysiology and record channel currents (refer Warton et al., 2002 or Singh et al., 2006)
4. In fig.1, please add DIDS and NPPB as controls.
5. In fig.3, please add IAA-94 for all pH experiments. It is not clear what percentage of Cl efflux at low pH is through CLIC1.
Also please include vesicles without CLIC1.

Minor issues
1. It is not clear why a high concentration of Zn was used in supplementary data (500 uM as opposed to 10 uM in other experiments).
2. Please add a reference to the lipid mix used in methods (Warton et al., 2002 and Singh et al., 2006).

First revision

Author response to reviewers' comments

I would firstly like to thank all the editorial team at Journal of Cell Science and the referees for supplying such a constructive critique of the work submitted for publication. As suggested, I have re-written the manuscript, guided by the feedback provided by the referees. Below I have provided a point by point response in blue to the referee’s comments in black.

© 2022. Published by The Company of Biologists under the terms of the Creative Commons Attribution License (https://creativecommons.org/licenses/by/4.0/).

REVIEWER REPORT(S): Referee: 1

Comments to the Author

The manuscript is overall well written and provides sufficient experimental data to support the findings. However, the following points need to be addressed prior to publication.

1. As others in the literature have also reported on alternate mechanisms for the membrane insertion of CLIC1, and there is likely to be more than one mechanism regulating this process, the abstract should be corrected to reflect this. A present the abstract presents these findings as exclusive, which is misleading. Suggested wording shown below:

"Molecular switches promoting CLIC1 activation remain under investigation. Here, cellular chloride efflux assays and immunofluorescence microscopy studies have identified Zn2+ intracellular release as a trigger for CLIC1 activation and membrane relocalisation. Biophysical assays confirmed specific binding to Zn2+, inducing membrane association and enhancing chloride efflux in a pH dependent manner. Together, our results identify a two-step mechanism with Zn2+ binding as a molecular switch promoting CLIC1 membrane insertion, followed by pH activation of chloride efflux."

We would like to thank this referee for their obvious support/interest in our work. We have updated the abstract as suggested to this version:

The CLIC protein family displays the unique feature of altering its structure from a soluble form to a membrane-bound chloride channel. CLIC1, a member of this family, is found in the cytoplasm or in internal and the plasma membranes, with membrane relocalisation linked to endothelial disfunction, tumour proliferation and metastasis. The molecular switch promoting CLIC1 activation remains under investigation. Here, cellular chloride efflux assays and immunofluorescence microscopy studies have identified intracellular Zn^{2+} release as the trigger for CLIC1 activation and membrane insertion. Biophysical assays confirmed specific binding to Zn^{2+}, inducing membrane association and enhancing chloride efflux in a pH dependent manner. Together, our results identify a two-step mechanism with Zn^{2+} binding as the molecular switch promoting CLIC1 membrane insertion, followed by pH activation of chloride efflux.

2. The following statement in the introduction should reference (Valenzuela et al., 2000) "CLIC1's integral membrane form has been found to be localised mostly in the nuclear membrane, although it is present in the membranes of other organelles and transiently in the plasma membrane."

We have added the reference as indicated.

3. Further to point 1, the following statement (and following paragraph) is also not entirely accurate and is simplistic in its argument: "Despite its clinical importance, to date very little and conflicting information is available for CLIC1 membrane insertion mechanism, and the structure of the channel form is unknown."

Yes, the precise structural details of the membrane form of CLIC1 remains unknown, however the following references shed light on its membrane insertion and/or membrane structure and there are increasing details regarding various mechanisms regulating its transition from soluble to membrane forms. In particular, the paper by Hossain et al,2017 (Langmuir) included below, provides detailed information regarding the membrane structure determined by X-ray and neutron reflectometry. These more subtle points should be reflected within the manuscript to indicate there is a complex and nuanced regulation processes for CLIC1 membrane insertion.

We have made the following changes to the text following this suggestion in the introduction:

Despite its clinical importance, to date only conflicting information is available on the mechanism of CLIC1 membrane insertion, and a high-resolution structure of the channel form has not been determined… Thus, long standing inconsistencies in the data surrounding the molecular switch that transforms CLIC1 from its soluble form into a membrane bound channel, and the

© 2022. Published by The Company of Biologists under the terms of the Creative Commons Attribution License (https://creativecommons.org/licenses/by/4.0/).

complex nature of its regulation, have prevented further advances in the understanding of CLIC1 function.

And in the discussion section:

While the structural rearrangements involved in this process are not yet fully understood, the molecular switch between the soluble and membrane bound forms is now elucidated, complementing the previously known effects of pH and cholesterol.

In addition, we have added the reference *KR Hossain, SA Holt, AP Le Brun, H Al Khamici, SM Valenzuela "X- ray and neutron reflectivity study shows that CLIC1 undergoes cholesterol-dependent structural reorganization in lipid monolayers" Langmuir 33 (43), 12497-12509*

REVIEWER REPORT(S): Referee: 2

Chloride Intracellular Ion Channel proteins are a novel class of ion channels. One of the CLIC proteins, CLIC1 preferentially localizes to intracellular organelles but on overexpression, they translocate to the plasma membrane. It is not clear how CLICs localize to membranes from the cytosol and whether they conduct Cl through membranes. Authors have shown that Zn not Ca is important for translocation of CLIC1 to the membrane and also increases Cl conductance. The manuscript is important for the field and will influence future work on CLICs.

Comments to the Author

The manuscript is focused on understanding the mechanism involved in the localisation of CLIC1 to the membrane. The data presented by the authors to support the role of Zn in CLIC1 to the membrane is convincing but the functional data is not promising.

We would like to extend our thanks to this referee for their attention to detail and interest in our work. In responding to all the comments above, we feel that we have improved the communication of our work and hope that it is now acceptable for publication in Journal of Cell Science.

I have the following comments.

1. *CLIC1 is known to localise to the nuclear membrane. In fig. 2, the authors should show colocalization with nuclear and plasma membrane markers. Colocalisation should be quantified.*
2. *In Fig 2. experiments should also be carried out in presence of ionophore as done for other experiments.*

We thank the reviewer for these suggestions. We have now collected new fluorescence microscopy images in HeLa and Glioblastoma cells in the presence of the ionophore Ionomycin with and without the Zn^{2+} chelator TPEN (New figure 2) adding a membrane stain. While quantification was not possible due to the permeabilization needed to stain for CLIC1, these images show clear membrane localization of CLIC1 upon addition of ionomycin.

© 2022. Published by The Company of Biologists under the terms of the Creative Commons Attribution License (https://creativecommons.org/licenses/by/4.0/).

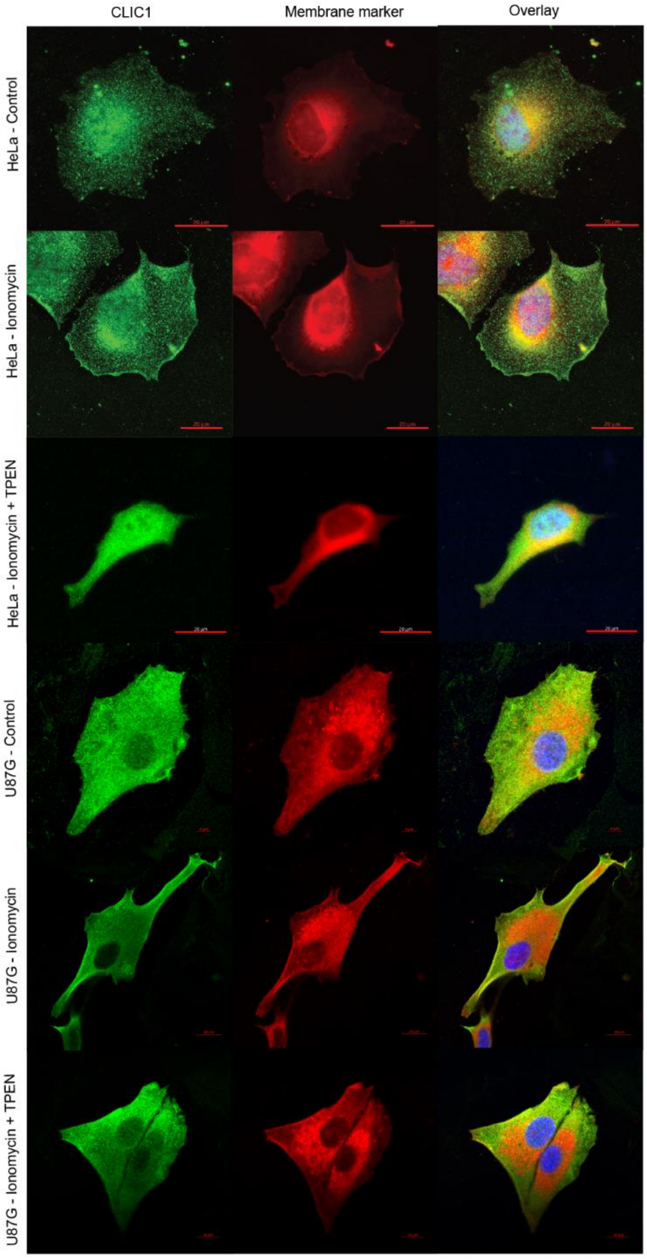

Figure 2: Divalent cations promote membrane insertion. CLIC1 localisation in Hela and U87G cells. HeLa and Glioblastoma were either untreated (1st and 4th row), treated for 3 hours with 10 μM Ionomycin (2nd and 5th row) or treated with 10 μM Ionomycin and the Zn^{2+} chelator TPEN (3rd and 6th row). CLIC1 typically exists in the cytosol and the Ionomycin-driven release of Ca^{2+}/Zn^{2+} promoted the presence of CLIC1 at plasma membrane. This effect is reversed upon chelation of intracellular Zn^{2+} by TPEN. Samples were stained with a CLIC1 antibody (green), a CellMASK membrane dye (red) and DAPI (blue). Scale bars indicated in all images correspond to 5 - 20 μm.

We have updated the results section describing these results accordingly:

In light of these results, we questioned if Zn^{2+} could trigger CLIC1 membrane relocalisation in cells. Endogenous CLIC1 localisation was monitored in HeLa and Glioblastoma (U87G) cells using immunostaining. CLIC1 typically exists in the cytosol in untreated Hela cells, and the addition of the ionophore Ionomycin promoted the presence of CLIC1 at the plasma membrane (Figure 2). A similar effect was observed in U87G cells. Addition of Ionomycin increased the degree of CLIC1 plasma membrane localisation. For both cell lines this effect was reversed by the addition of TPEN, confirming that Zn^{2+} triggers the activation and membrane relocalisation of CLIC1.

© 2022. Published by The Company of Biologists under the terms of the Creative Commons Attribution License (https://creativecommons.org/licenses/by/4.0/).

3. The major issue with proposing CLICs as functional ion channels is the time taken for Cl efflux. This is a long-standing argument in the field. In figure 1, it will be helpful to show fluorescence vs time. If CLIC1 is an ion channel, one should observe a faster component in the graph. Alternatively, you can perform electrophysiology and record channel currents (refer, Warton et al., 2002 or Singh et al., 2006)
4. In fig.1, please add DIDS and NPPB as controls.

We thank the reviewer for this observation around the argument in the field over CLIC1 activity as a chloride channel. In this paper we are not trying to explore the chloride channel activity. Instead, we have focused on the activation mechanism, and we confirm its effect on the chloride channel activity replicating published assays in the CLIC1 field with very similar results (Tulk et al, 2000).

As suggested, we have included a plot of fluorescence vs time in Supplementary figure 1.

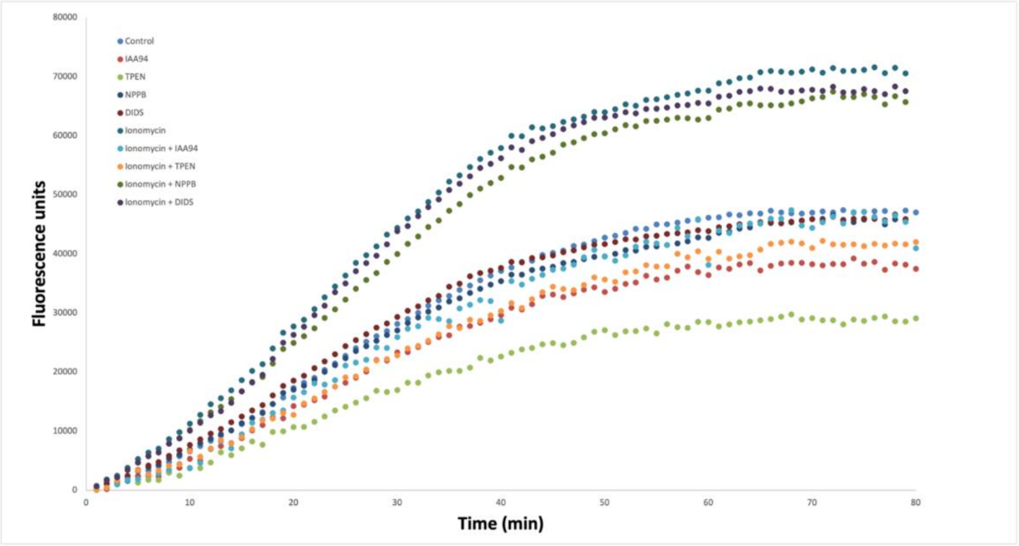

Figure S1 - Effect over time of different treatments on chloride efflux (measured as fluorescence intensity units of the dye MQAE) in MQAE-stained U87 cells exposed to IAA94 (10 μM), TPEN (5 μM), NPPB, DIDS and Ionomycin (10 μM) for 80 min. Values constitute means of six independent determinations.

The rates observed here are not solely dependent on the efflux rates of CLIC1 as the time course starts upon addition of ionomycin. Therefore, the kinetics of action of ionomycin, membrane relocalisation of CLIC1 and its activation must be taken into consideration in the overall rates of efflux measured in this experiment.

Following this reviewer’s comment, we have also collected chloride efflux experiments in the presence of the inhibitors DIDS and NPPB in U87G cells in the absence and presence of Ionomycin (new Figure 1). As observed previously (warton et al, 2002), we didn’t detect CLIC1 selective inhibition of its chloride channel by DIDS or NPPB.

© 2022. Published by The Company of Biologists under the terms of the Creative Commons Attribution License (https://creativecommons.org/licenses/by/4.0/).

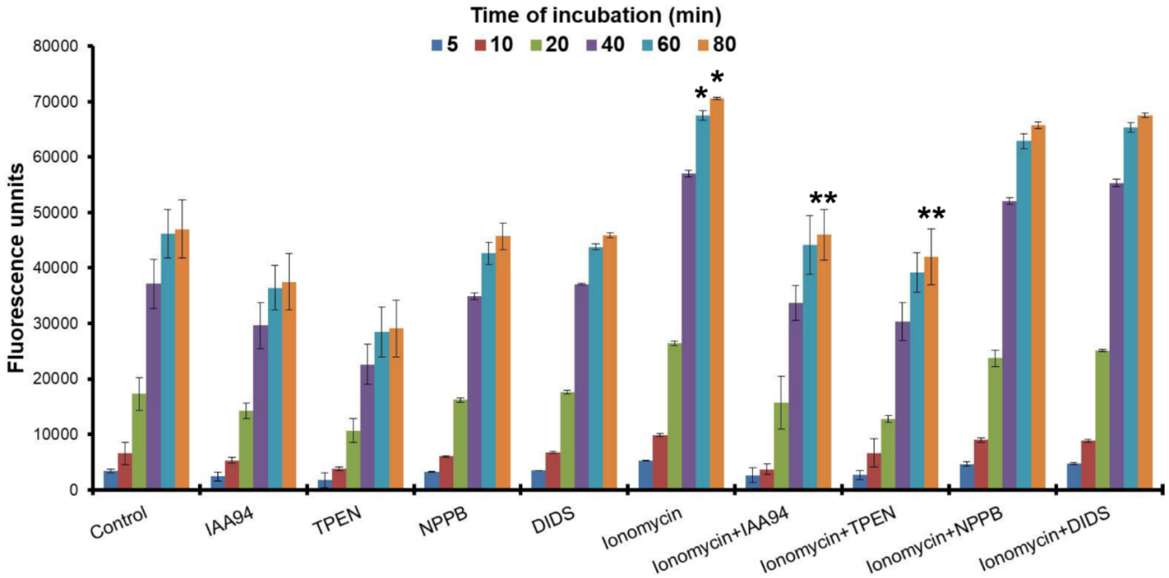

Figure 1. Effect of different treatments on chloride efflux (measured as fluorescence intensity units of the dye MQAE) in MQAE-stained U87 cells exposed to IAA94 (10 µM), TPEN (5 µM), NPPB, DIDS and/Ionomycin (10 µM) for 80 min. Values constitute means of six independent determinations ± standard deviation. Ionomycin treatment resulted in statistically significant differences from 60 min compared to the control samples ($p=0.0123$) (* $P < .05$) and to all other treatments (** $p<.005$) ($n=6$).

We have updated the results section accordingly:

The effect of divalent cation-driven membrane localisation on the chloride efflux activity of CLIC1 was tested in U87G cells using MQAE as a fluorescent reporter of the intracellular concentration of Cl^-. With no treatment, the chloride efflux activity is not affected by the chloride channel inhibitors DIDS and NPPB, in line with previous results (Warton et al., 2002a), and is only modestly inhibited by the CLIC inhibitor IAA-94 or the Zn chelator TPEN, and thus is likely not CLIC mediated. Ionomycin, an ionophore shown to increase both Ca^{2+} and Zn^{2+} intracellular concentrations, induced a significant increase ($p=.0123$) of the Cl- efflux, as seen by the increase in MQAE fluorescence, that was reversed upon treatment with the Zinc chelator TPEN or with the CLIC inhibitor IAA-94 ($p<.005$) (Xu et al., 2016) (Figure 1). Interestingly, TPEN inhibits the chloride conductance of the control cells to a level similar to IAA-94, suggesting the involvement of Zn^{2+} in the activation of CLIC1 chloride efflux activation. Again, DIDS and NPPB showed no effect on CLIC1 efflux.

5. In fig.3, please add IAA-94 for all pH experiments. It is not clear what percentage of Cl efflux at low pH is through CLIC1. Also please include vesicles without CLIC1.

We apologise for not making the description of this experiments clearer. This experiment was performed in vesicles incubated only with CLIC1. Therefore, any chloride conductance can only be due to CLIC1, as there was not any other component in the system. Our results match very well those presented in (warton et al, 2002 & Tulk et al, 2000), which also showed IAA94 inhibition of chloride efflux. In light of this, we don't believe that the addition of IAA94 is relevant in this specific experiment.

In addition, the data is presented as rates above control (vesicles without protein) rates. Therefore, we are already considering the vesicles without CLIC1 as a control.

Minor issues

1. It is not clear why a high concentration of Zn was used in supplementary data (500 uM as opposed to 10 uM in other experiments).

© 2022. Published by The Company of Biologists under the terms of the Creative Commons Attribution License (https://creativecommons.org/licenses/by/4.0/).

The difference between Zn concentrations relates to the type of assay. In-cell experiments were conducted at 10 μM Zn^{2+}, while in-vitro experiments with purified protein use a 10fold molar excess of Zn^{2+} to ensure full insertion in the membrane.

2. *Please add a reference to the lipid mix used in methods (Warton et al., 2002 and Singh et al., 2006).*

We have now added the relevant reference in the methods section: Tulk, B.M., Kapadia, S., and Edwards, J.C. (2002). CLIC1 inserts from the aqueous phase into phospholipid membranes, where it functions as an anion channel. Am. J. Physiol. - Cell Physiol. *282*, 1103–1112.

<u>Second decision letter</u>

MS ID#: JOCES/2021/259704

MS TITLE: Zn^{2+} triggered two-step mechanism of CLIC1 membrane insertion and activation into chloride channels

AUTHORS: Lorena Varela, Alex C Hendry, Joseph Cassar, Ruben Martin-Escolano, Diego Cantoni, Felipe Ossa, John C Edwards, Vahitha Abdul-Salam, and Jose Luis Ortega-Roldan
ARTICLE TYPE: Research Article

I am happy to tell you that your manuscript has been accepted for publication in Journal of Cell Science, pending standard ethics checks.

<u>Reviewer 1</u>

Advance summary and potential significance to field

Revisions made by the authors have addressed my previous comments.

Comments for the author

N/A

<u>Reviewer 2</u>

Advance summary and potential significance to field

The manuscript provides an important mechanism in membrane insertion of intracellular ion channel.

Comments for the author

Authors have addressed all my concerns. I still believe Zn2+ might be promoting insertion into the nuclear membrane which should be reported. The membrane marker in the figure is not very clear for the nuclear membrane.
Apart from this minor concern, I thank authors to address concerns raised as it is vital for the CLIC field.

© 2022. Published by The Company of Biologists under the terms of the Creative Commons Attribution License (https://creativecommons.org/licenses/by/4.0/).
